# Hsp70 diversification and repurposing across the tree of life: Lessons from the evolutionary and mechanistic trajectory of the Hsp70–Hsp110 chaperone system

**DOI:** 10.1111/febs.70535

**Published:** 2026-04-14

**Authors:** Pierre Goloubinoff, Paolo De Los Rios, Mathieu E. Rebeaud

**Affiliations:** ^1^ Department of Plant Molecular Biology, Faculty of Biology and Medicine University of Lausanne Switzerland; ^2^ Institute of Physics, School of Basic Sciences École Polytechnique Fédérale de Lausanne – EPFL Lausanne Switzerland; ^3^ Institute of Bioengineering, School of Life Sciences École Polytechnique Fédérale de Lausanne – EPFL Lausanne Switzerland

**Keywords:** evolution, Hsp110, Hsp70, NEF, structure–function

## Abstract

The 70 kDa Heat Shock Proteins (Hsp70s) are molecular chaperones ubiquitous in most free‐living bacteria and archaea, and in the cytosol and ATP‐containing organelles of eukaryotic cells, where they act as ATP‐fueled nanomachines that unfold, remodel, and translocate polypeptides. In bacteria, Hsp70 (DnaK) operates with the J‐domain cochaperone DnaJ and the nucleotide exchange factor (NEF) GrpE, which accelerates ADP release and ATP rebinding. Many simple prokaryotes encode a single canonical DnaK, but more complex bacteria often harbor additional DnaK‐derived paralogues with modified, truncated, or missing domains required for substrate and DnaJ binding, raising questions about their functions and possible roles in proteostasis. A suggestive parallel comes from eukaryogenesis, when a gene duplication of an ancestral Hsp70 gave rise to Hsp110. Hsp110s have largely abandoned direct DnaJ, GrpE, and substrate binding; yet, through the reversible formation of NBD‐mediated Hsp70–Hsp110 heterodimers, they act as potent NEFs that enhance Hsp70‐driven disaggregation of compact protein aggregates. By analogy, we propose that NBD‐containing DnaK paralogues in prokaryotes may likewise function as dedicated DnaK cochaperones, tuned to boost protein disaggregation and repair and specialized for the specific lifestyles and associated stressors of these organisms.

## Graphical abstract

Evolutionary and mechanistic divergence in the Hsp70–Hsp110 chaperone system. Prokaryotic Hsp70s probably diversified into multiple orthologues that cooperated with co‐chaperones such as JDPs and NEF, forming increasingly complex proteostasis networks. In eukaryotes, gene duplication and structural repurposing diverged into Hsp110 which lost its main unfoldase activity and evolved into a NEF that cooperates with Hsp70 to refold aggregated proteins and participate in specialized functions (Representative organisms' pictures from https://www.phylopic.org).

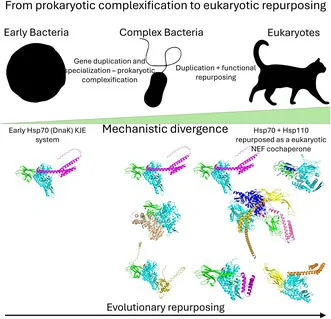

**Abbreviations**
ADP: Adenosine Diphosphate
ATP: Adenosine Triphosphate
BiP: Binding immunoglobulin Protein
GRP170: Glucose‐Regulated Protein 170
Hsp110: 110 kDa Heat Shock Proteins
Hsp70: 70 kDa Heat Shock Proteins
JDP: J‐Domain Protein
LECA: Last Eukaryotic Common Ancestor
NBD: Nucleotide Binding Domain
NEF: Nucleotide Exchange Factor
SBD: Substrate Binding Domain

## Introduction

The first simple organisms in Earth's primitive oceans likely contained short single‐domain proteins enriched with *α*‐helices [1]. As biological functions diversified, distinct single‐domain polypeptides were increasingly assembled into oligomeric complexes [2]. In bacteria, such complexes are often encoded on polycistronic operons, which can promote coordinated expression and cotranslational association of subunits synthesized on nearby ribosomes [3]. In parallel, multidomain polypeptides emerged [4, 5], providing a different, potentially more efficient strategy to build complex architectures within a single polypeptide chain. However, both oligomerization and multidomain proteins often introduce large exposed hydrophobic interfaces in the folding and assembly intermediates and further steps that must be tightly coordinated in time and stoichiometry, increasing the risk of misfolding and aggregation, especially under stress, such as heat shock. Moreover, in the course of their evolution, proteins increased their *β*‐strand content [1]. Partially unfolded *β*‐strand‐rich regions are generally more prone than *α*‐helices to form non‐native *β*‐structures, which upon self‐assembly, can give access to a generic, amyloid‐like state that many unrelated sequences can adopt [6]. These non‐native *β*‐assemblies can nucleate the growth of insoluble, inactive oligomers and higher‐order aggregates [1].

Protein misfolding and aggregation can cause loss of specific biological functions and, additionally, toxic gain of function, because exposed hydrophobic surfaces on aggregates can interact improperly with other proteins, globally perturbing the proteome, and with membranes, altering their permeability and fluidity [7, 8, 9]. As proteins and complexes became more elaborate during evolution, these increased risks of misfolding and aggregation were accompanied by the expansion of molecular chaperone networks and chaperone‐gated proteases that target protein aggregates, solubilize them and promote their refolding into native functional states, or degrade irreversibly damaged polypeptides to recycle their building blocks [10]. Hsp70 chaperones emerged as a central hub of these protein homeostasis machineries [11] and have diversified over billions of years into numerous homologs across all branches of the tree of life.

In this work, we briefly review the basic features of the Hsp70‐based unfolding/disaggregation system and its regulation by cochaperones, and we highlight the presence, in prokaryotes, of a vast diversity of derivatives of unknown function. We propose here that understanding Hsp110, the ubiquitous eukaryotic Hsp70 derivative that acts as a cochaperone of Hsp70, may offer clues and testable hypotheses on the functions of the prokaryotic Hsp70 derivatives.

## Structure and function of canonical Hsp70

**Fig. 1:**
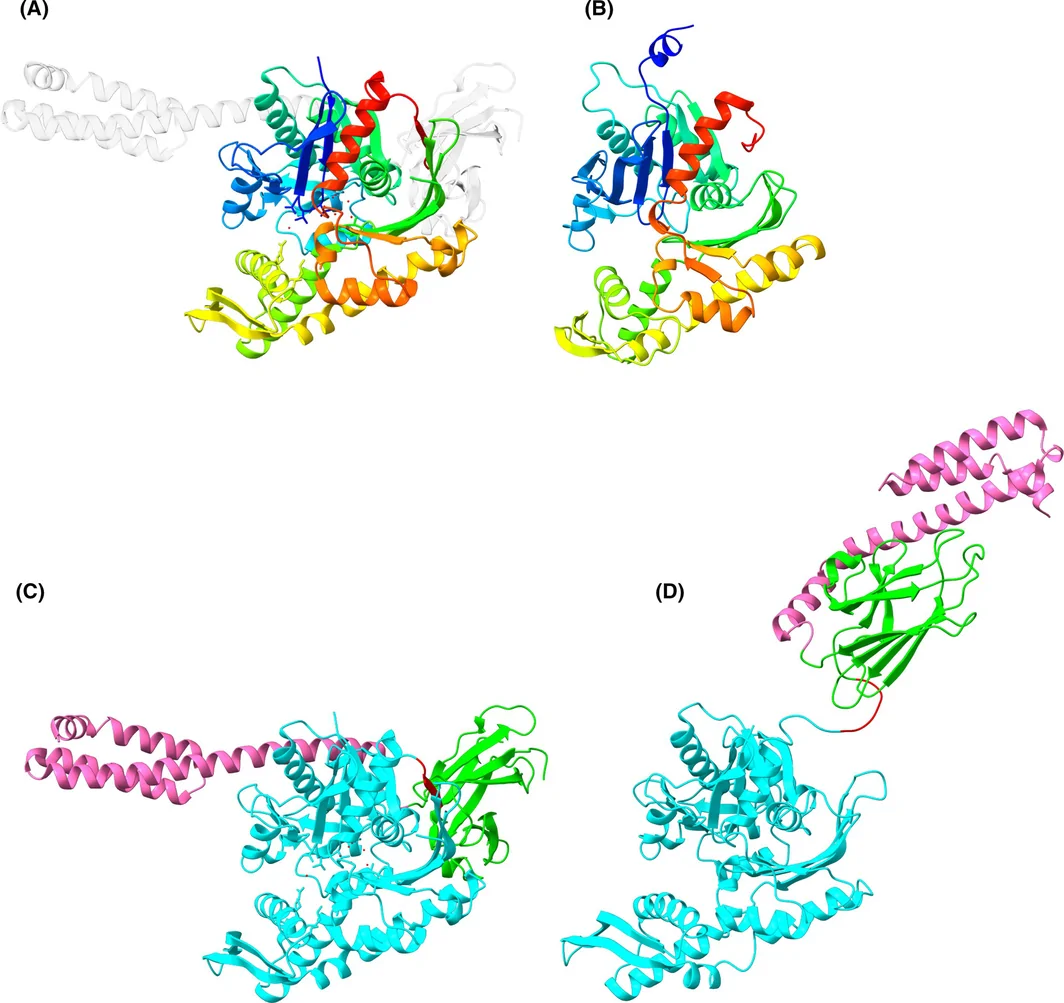
Structures of *E. coli* DnaK and MreB. (A) Crystallographic structure of *Escherichia coli* (*E. coli*) DnaK (PDB ID: 5NRO) [17], where only the Nucleotide Binding Domain (NBD) is not transparent to highlight its structure; (B) the structure of *E. coli* MreB (AF‐P0A9X4‐F1) predicted with AlphaFold [18]. In (A) and (B), the *α*‐helices and *β*‐sheets of the two domains are rainbow‐colored to ease appreciating the similarity of the two folds. (C) ATP‐bound DnaK (PDB ID: 5NRO), where the linker (red) is *β*‐structured and bound to the NBD (cyan), the lid (magenta) is bound to the NBD and separated from the basket (green). (D) ADP‐bound DnaK (PDB ID: 2KHO) [19], where the linker is unstructured and unbound from the NBD, the lid is separated from the NBD and tightly bound to the basket.

The Hsp70s are members of a very ancient group of primordial molecular chaperones that likely evolved in the first terrestrial bacteria about 3 billion years ago from an actin‐hexokinase common ancestor [12], and soon became acquired through horizontal gene transfer by most bacteria and archaea [1, 13, 14]. The Hsp70s are widely distributed across ATP‐containing subcellular compartments, such as the cytosol, endoplasmic reticulum, lysosome, mitochondria and chloroplasts [1, 11]. The domain organization of canonical Hsp70s is highly conserved from bacteria (where they are called DnaK) to mammals [15, 16]. It comprises the N‐terminal Nucleotide Binding Domain (NBD) (Fig. 1A), which shares high structural and some sequence similarity with *E. coli*'s actin, the cell shape‐determining protein MreB (Fig. 1B). The NBD (cyan in Fig. 1C,D), which binds ATP or ADP and where ATP hydrolysis takes place, is connected by a highly conserved linker (red, Fig. 1C,D) to the C‐terminal Substrate Binding Domain (SBD), which engages substrates that need to be remodeled by Hsp70. The SBD is in turn composed of two subdomains: a twisted *β*‐sandwich fold (green, Fig. 1C,D), which is commonly referred to as the *β*‐basket in the chaperone field, and an *α*‐helical lid (magenta, Fig. 1C,D). When the NBD is in complex with ATP, the SBD is open, with the *β*‐basket and the *α*‐helical lid separated and independently docked to the NBD, and the linker has *β*‐strand conformation and is docked in the NBD (Fig. 1C). In contrast, when the NBD is bound to ADP, both the SBD and the linker are undocked from the NBD, and the two SBD subdomains become closely bound to one another. In this conformation, the NBD and SBD remain connected only by means of the unstructured flexible linker (Fig. 1D).

The full activation of Hsp70's ATPase requires binding of both a J‐domain protein (JDP) cochaperone and the polypeptide substrate [20]. JDPs (DnaJ being the most widespread in bacteria) are defined by their namesake J‐domain, a ~65‐residue module that contacts all Hsp70 structural elements, namely the NBD, the SBD, and the linker. In particular, 99% of all wild‐type J‐domains have a highly conserved His‐Pro‐Asp (HPD) motif [21] that is necessary to stimulate ATP hydrolysis by means of specific contacts with the Hsp70 linker. Instead, the polypeptide substrate stimulates hydrolysis through a different allosteric pathway, triggered by binding to the *β*‐basket of the SBD [22, 23]. Beyond the J‐domain, JDPs carry additional modules that bind and localize substrate proteins such that the J‐domain and the substrate engage Hsp70 simultaneously, boosting its ATPase activity by two to three orders of magnitude [20, 24].

Upon concerted stimulation of ATP hydrolysis by both a JDP and the colocalized substrate, typically a misfolded or natively folded polypeptide segment, the major rearrangement of the SBD subdomains (Fig. 1C,D) leads to substrate trapping. This dynamic process converts the energy of ATP hydrolysis into an enhanced nonequilibrium affinity (‘ultra‐affinity’) for the substrate [25]. This is causing the substrate, if not already an extended segment, to unfold in order to fit within the closed SBD pocket. Binding of one or more Hsp70 molecules onto nearby misfolded segments from the same polypeptide may then promote progressively to the complete unfolding and even the stretching of the polypeptide substrate by entropic pulling [26, 27, 28]. The chaperone cycle completes when nucleotide exchange factors (NEFs, GrpE in bacteria) catalyze ADP release and the reopening of the SBD (Fig. 1C,D) with the consequent release of the unfolded intermediate, which may then refold to its native conformation with no affinity for the chaperone. The action of canonical Hsp70s thus stringently relies on four features: (i) its structural elements (NBD, SBD, and linker), (ii) its obligatory interaction, in the ATP‐bound state, with J‐domains, (iii) the interaction of its SBD with polypeptide substrates, and (iv) its ATPase activity. *In vitro* experiments show that the interaction with NEFs, while important, is less crucial as in the absence of GrpE, stable protein aggregates can be efficiently solubilized and refolded by solely DnaJ, DnaK, and ATP, albeit more slowly than in the presence of GrpE [29, 30].

Through this fundamental cycle, Hsp70s act as ATP‐powered unfolding nanomachines that, by stretching misfolded polypeptides [27, 30, 31, 32, 33, 34], can bring them back to the native folding pathway and can prevent and actively revert the stress‐induced formation of inactive and potentially toxic protein aggregates [35, 36, 37, 38, 39]. Through the same molecular mechanism, organellar Hsp70s can also drive the unfolding and the unidirectional import into the ER [40], the mitochondria [26, 41] and chloroplasts [42] of organellar polypeptides that are synthetized on cytosolic ribosomes. Hsp70s also contribute to regulate the proper assembly of functional oligomers [43, 44, 45] and may facilitate the controlled degradation of misfolded proteins by chaperone‐gated proteases [46, 47]. Furthermore, many Hsp70 genes are overexpressed under various stresses, heat in particular, underscoring their fundamental role in cellular protein homeostasis, both under physiological conditions and in response environmental stresses [48, 49].

## Prokaryotic DnaK and DnaK derivates

While whole genome analysis reveals that some prokaryotes, predominantly Archaea with small genomes, can thrive in the absence of Hsp70 [50], most prokaryotes with more complex proteomes harbor one or more genes for DnaK. The average number of genes identified as DnaK‐related in a list of 130 prokaryotic genomes [1] (see Data S1) was 2.1 ± 2.1 (2.3 ± 2.3 excluding Archaea) hinting at the presence of several DnaK paralogs per organism. It should be noted that since the publication of our previous study, the classification of certain proteomes has either been removed from Uniprot or reclassified as redundant (~20 out of 130). However, upon verification with the current proteomes, this does not significantly alter the results of our analysis. In some extreme cases, the genome contained up to 15 DnaK‐related genes. To provide a more intuitive view, we selected and analyzed the paralogs from four bacteria, namely *Pichrophilus torridus* (1 DnaK gene), *Escherichia coli* (4), *Anaeromyxobacter dehalogenans* (12) and *Myxococcus Xanthus* (15). In the case of the well‐studied model prokaryote *E. coli*, the four Hsp70‐like genes are the conserved archetypical DnaK; two close but clearly diverging homologs of DnaK, namely HscA, specialized to assist the assembly of iron sulfur clusters [51], and HscC, an Hsp70 thus far reported to confer resistance to Cd^2+^‐ions and UV irradiation and capable of partially prevent protein aggregation [52], both with shortened *α*‐helical lids [53]; and YegD, which consists only of the DnaK‐like NBD devoid of linker, basket and lid.

**Fig. 2:**
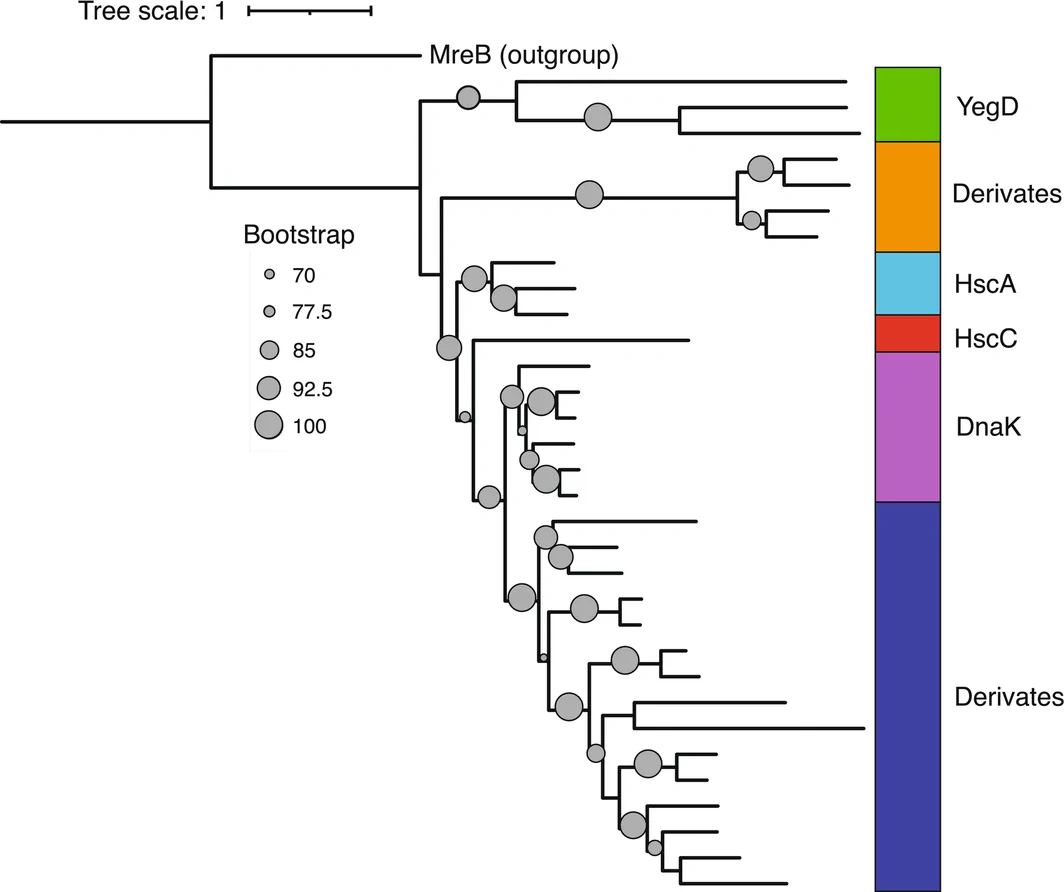
Phylogenetic tree of the DnaKs and derivate proteins in four selected prokaryotes. Maximum Likelihood Phylogenetic tree (bootstrap values in gray circles) of the selected 32 sequences with color bands based on the groups of proteins: YegD (green). Canonical DnaK (magenta), HscA (cyan), HscC (red) and NBD‐containing DnaK Derivates (two phylogenetically separate groups, orange and blue).

**Fig. 3:**
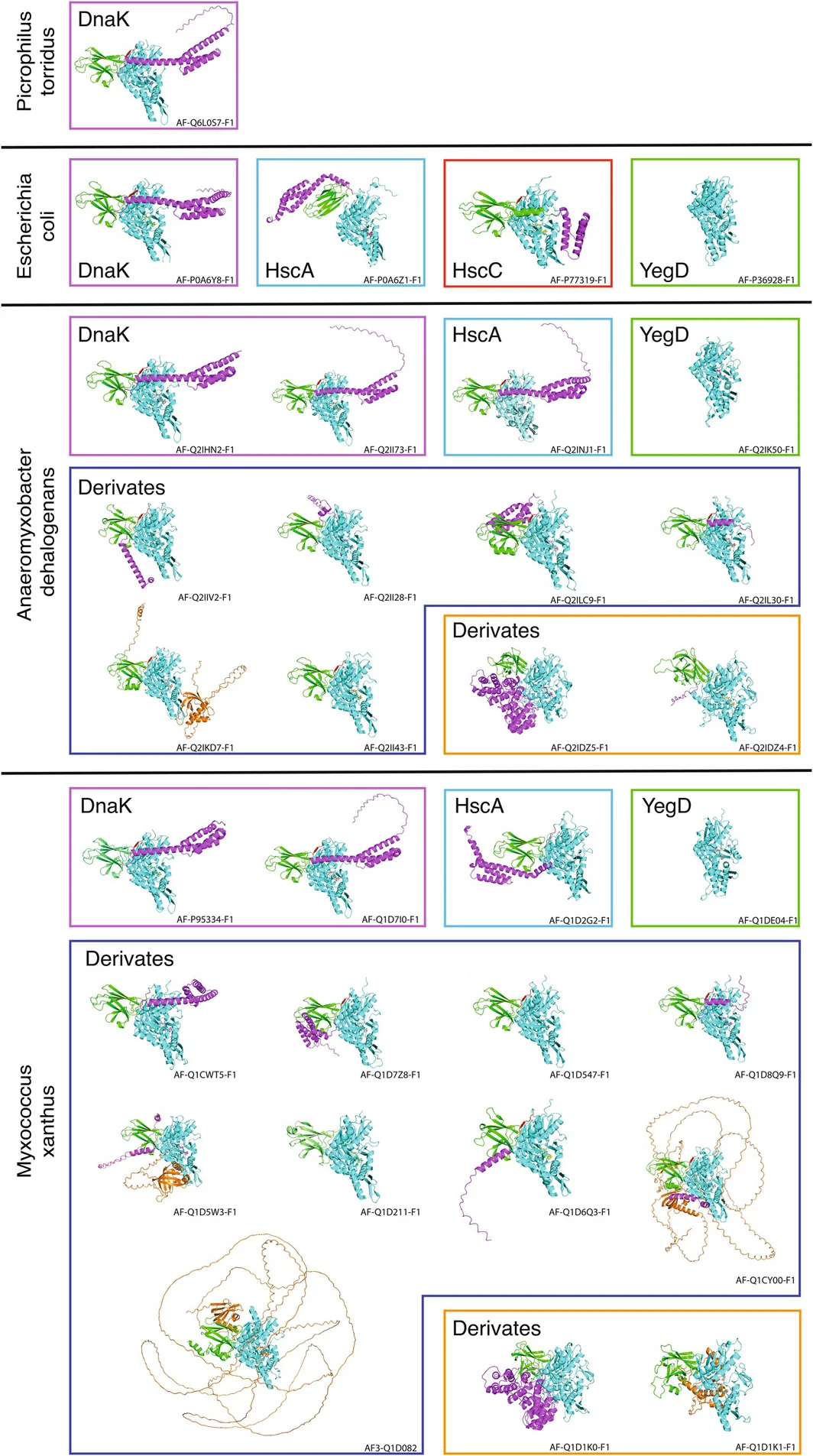
AlphaFold predicted structures for all the DnaKs and derivatives proteins in four selected prokaryotes. The different groups have been enclosed in boxes colored according to the scheme in Fig. 2 (Magenta box for DnaK, Cyan box for HscA, Red box for HscC, Green box for YegD, Blue and Orange boxes for the two phylogenetically distinct groups of Derivates). When identifiable, the different domains are colored according to the scheme in Fig. 1C,D (Nucleotide Binding Domain (cyan), linker (red), lid (magenta) and basket (green)), with additional N‐ and C‐terminal domains in orange. Models are AlphaFold prediction [18].

A phylogenetic tree of the DnaK‐related genes of these bacteria (including *E. coli* MreB as an outgroup) clearly clusters some of them together and coherently with the *E. coli* genes (Fig. 2). This analysis proceeds best in tandem with a structural analysis, using AlphaFold predictions [18], which should be interpreted based on the pLDDT, pTM, and ipTM values (see Data S1) (Fig. 3). For example, the genes phylogenetically classified as a *bona fide* DnaK (magenta in Figs 2 and 3) have a prototypical NBD, a highly conserved and easily recognizable linker (^388^DVLLLD^393^ in *E. coli*) and a classic SBD comprising the basket and the lid SBD. Moreover, we find that these *true* DnaK (K) genes are always present together with genes encoding for at least one DnaJ (J) and GrpE (E) cochaperones (Data S1), which can be arranged in the same operons, but can also be clustered in several different places in the genome, each encoding for parts of the system, where the distribution can be tripartite or bipartite, that is, KEJ, JKE, KE, and KJ [54]. Similarly, there are groups corresponding to YegD (green) and to HscA (cyan), and the corresponding proteins grouped together have similar structures. To the extreme of the spectrum, the genome of *M. xanthus* encodes for two canonical DnaKs [55], one HscA, one YegD and 11 more genes encoding for a conserved DnaK‐like NBD (‘Derivates’ in Figs 2 and 3, blue), only one of which being predicted to be structurally similar to canonical DnaKs (Fig. 3). The remaining genes lack part of the SBD (shortened or deleted lid) or its entirety (both lid and basket) are missing, with often additions of structured or unstructured domains of unknown function in the N or C termini (Data S1). The repertoire of DnaK‐related genes of *A. dehalogenans*, which belongs to Myxococcales, encodes for 8 DnaK‐derivates, some being phylogenetically and structurally similar to those of the related *M. xanthus* (e.g., the yellow Derivates), and others being different (Fig. 3). These examples show just a small fraction of the staggering diversity of DnaK‐related proteins that can be found, for example, exploring the UniProt [56] database.

Since in all the analyzed prokaryotic genomes that contained only one NBD of DnaK, it was found to always be a *bona fide* DnaK gene and that no derived NBD paralogs were found in genomes lacking a *bona fide* DnaK gene, it is reasonable to suggest that the canonical DnaK represents a likely ancestral form from which other variants later evolved and diversified in some more complex prokaryotes. These derivates possibly emerged and were retained because they fulfilled specialized functions adapted to the distinct ecological niches and environmental constraints of those organisms. At the same time, the partial or complete lack of the canonical subdomains of the SBD, or its significant alteration, as well as the highly modified sequences of the linkers, and even the complete absence of linkers, suggest that many DnaK derivates lost the original ability of their putative DnaK ancestor to bind and be activated by misfolded polypeptides and by JDPs, while still possibly being able to hydrolyze ATP. These derivates would thus lack the ability to fulfill all the requirements to act as a canonical Hsp70. While this calls for a systematic experimental analysis of the ATPase, J‐domain‐, GrpE‐binding, as well as substrate‐binding and substrate‐processing abilities of the prokaryotic NBD‐derivates, for example, of the 2–3 DnaKs and the 12–13 DnaK‐like derivates that are encoded by *Myxococcus Xanthus*, it also raises a more fundamental question: What might be the molecular functions carried out by each distinct DnaK derivate and what could be their roles, independent or in synergy with the canonical DnaK‐DnaJ‐based protein homeostasis machinery, found to be apparently always present in the same genome?

## Hsp110 and Hsp70: Similar, but highly differentiated

Eukaryotic genomes provide an intriguing clue: a gene‐duplication event around two billion years ago, during eukaryogenesis, when the ancestor of eukaryotic Hsp110 cochaperones diverged from the much older, originally prokaryotic Hsp70 family.

Hsp110 proteins are large Hsp70 homologs that function as nucleotide exchange factors for cytosolic Hsp70s, while the ER paralog GRP170 serves the same role for the ER‐resident Hsp70 BiP [57, 58, 59, 60]. In contrast to canonical Hsp70s, which are broadly distributed across bacteria, archaea, and eukaryotes, Hsp110 family members appear to be restricted to eukaryotes, consistent with an origin linked to eukaryogenesis and the ensuing expansion and compartmentalization of proteostasis networks [1, 61]. Hsp110 activity was first characterized as heat‐inducible 110 kDa proteins in mammalian cells, including Chinese hamster ovary cells and rodent fibroblasts, and in nucleoli [62, 63, 64, 65, 66]. The corresponding genes were subsequently cloned from mammals, budding yeast, and sea urchins [67, 68, 69, 70]. Around the same period, targeted searches in plants, particularly in the model species *Arabidopsis thaliana*, extended this conservation to the plant lineage [71]. These results confirmed Hsp110 as a conserved eukaryotic chaperone family.

Given its absence in all prokaryotic genomes analyzed to date, Hsp110 seems to be a phylogenetic derivate of the first eukaryotic Hsp70 gene that was present in the Last Eukaryotic Common Ancestor (LECA), which itself likely originated from a Gram‐negative DnaK ancestor [1, 72, 73]. Sequence‐ and structure‐wise, Hsp110s have diverged from Hsp70s, and they are easily recognizable in simple multiple‐sequence alignments, in particular through their highly diverged and not overly conserved linker [74], the presence of long insertions and extensions in the *β*‐basket and in the *α*‐helical lid. Furthermore, the sequences of Hsp110s show greater sequence variation in the SBD than in the NBD, which is relatively conserved.

**Fig. 4:**
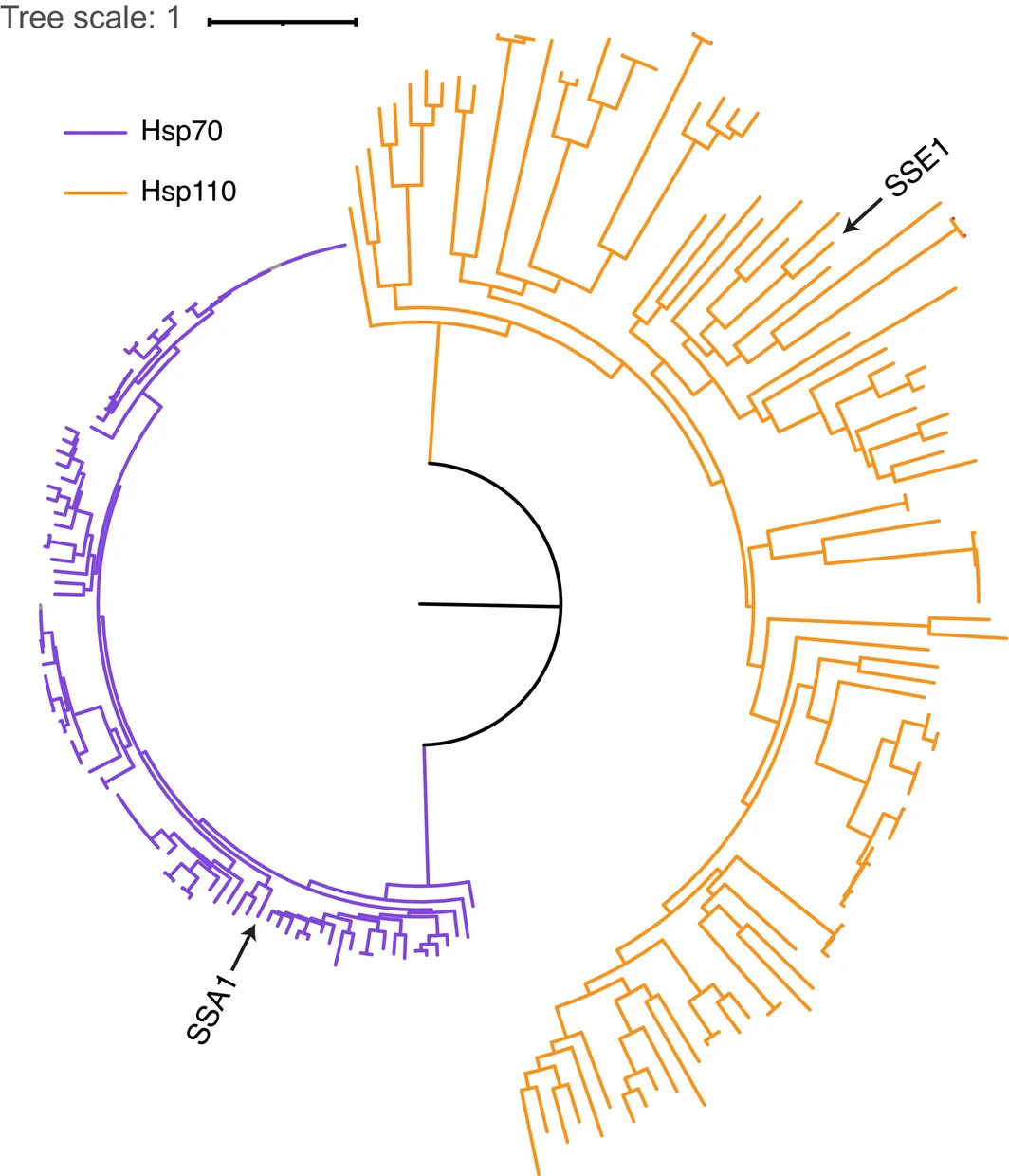
Common Maximum Likelihood phylogenetic tree of Hsp70 and Hsp110 in newly sequenced Protists. The first 100 top hits of Hsp70 (purple) and Hsp110 (orange) after a BLAST [78] with Ssa1 and Sse1 as queries against the 10′000 Protists Genome Project [75]. These results demonstrate that the Hsp110 sequences diverge more from each other than the ancestral Hsp70 sequences, which are instead highly conserved across all kingdoms [79] including protists, which are considered as the simplest eukaryotes.

A complementary perspective on the evolution of Hsp70 and Hsp110, and on their possible common origins, comes from the Protist 10 000 Genomes Project [75], which surveys diverse protist lineages, eukaryotes that are neither plants, animals nor fungi and that include several early‐branching eukaryotic clades [76]. From this database, we extracted 100 Hsp70 and 100 Hsp110 sequences, using yeast Ssa1 and Sse1 as reference queries. As expected, given this selection, the resulting phylogenetic tree (Fig. 4) clearly resolves two major clades corresponding to Hsp70 and Hsp110. Notably, the branches within the Hsp110 clade tend to be longer than those within the Hsp70 clade, consistent with greater sequence divergence among Hsp110s, due in particular to the greater variability of the SBD than of the NBD [77].

These new databases about simpler, possibly more primitive eukaryotes should enable a clearer census of Hsp70 and Hsp70‐like families, including Hsp110s, and help pinpoint when Hsp110 emerged after eukaryotisation. Moreover, the rapidly growing set of archaeal genomes, especially Promethearchaeati (ASGARD) [80], offers a particularly promising and complementary window into additional, potentially overlooked derivatives [81]. A key open question is what functional consequences followed the sequence and structural divergence of eukaryotic Hsp70 derivatives.

## Function(s) of Hsp110

Extensive experimental evidence for the role of Hsp110 proteins as nucleotide exchange factors raises several central questions. Why did evolution repurpose a duplicated Hsp70 toward this new NEF function, given that in prokaryotes it is already efficiently fulfilled by GrpE? In eukaryotes, GrpE remains nuclear encoded and thus necessarily transits through the cytosol on its way to mitochondria and, in plants, chloroplasts, yet no dedicated cytosolic GrpE isoform has been retained to serve, again as NEF to the cytosolic Hsp70s. When and why did this restriction to organelles arise? When did other eukaryote‐specific NEFs, such as armadillo‐like proteins (Fes1 in yeast, HspBP1 in human) [82] and BAGs (Bcl‐2–associated athanogene proteins) [1, 83], appear, did they precede or follow the emergence of Hsp110s and how may they complement, synergize, or compete with the Hsp110s?

Remarkably, besides its NEF activity, Hsp110 proteins play a crucial role in enhancing the disaggregation activity of the Hsp70–JDP system, with an efficiency unmatched by other NEFs, and acting on both amorphous and compact, fibrillar aggregates [36, 37, 84, 85, 86]. Several not mutually exclusive mechanisms have been proposed for this effect, all relying on a direct, albeit transient, interaction within the Hsp70‐Hsp110 heterodimer. These include recycling of ADP‐bound, inactive Hsp70s [39] and an amplification of entropic pulling forces arising from the larger size of the heterodimer [77]. This interaction primarily involves contacts of the Hsp110 NBDs with the NBD of Hsp70 precisely at the contact lobes where GrpE binds the NBD of DnaK [87].

**Fig. 5:**
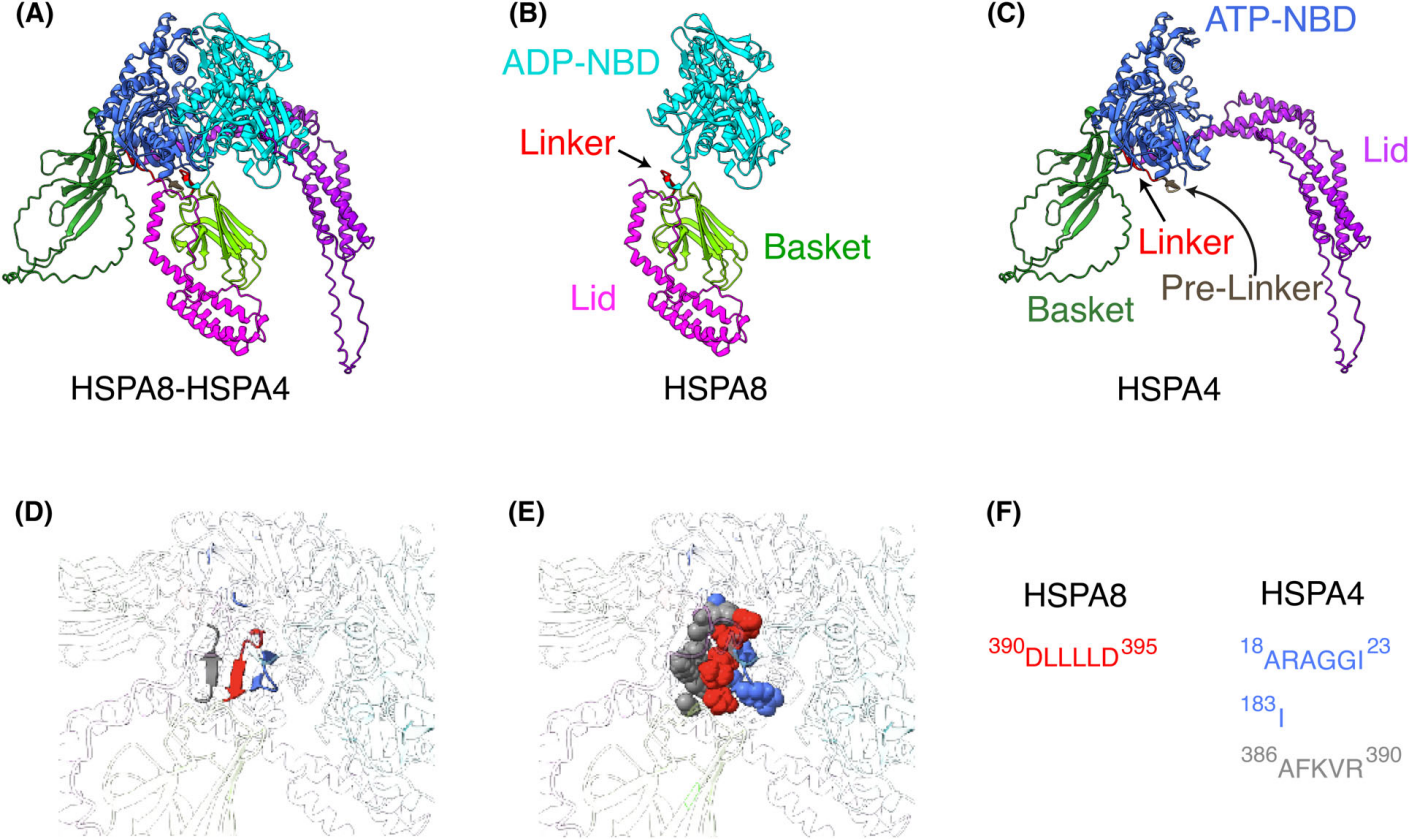
Structural prediction of the Hsp70‐Hsp110 heterodimer. (A) Structure of the complex of bovine Hsp70 (HspA8) and Hsp110 (HspA4); (B) Structure of HspA8 in the heterodimer, with main structural features indicated (Nucleotide Binding Domain (cyan), linker (red), lid (magenta) and basket (green); (C) Structure of HspA4 in the heterodimer, with main structural features indicated (Nucleotide Binding Domain (dark blue), linker (red), lid (purple) and basket (dark green); (D) Closeup of the Hsp70 linker (red) – Hsp110 prelinker (gray) arrangement, where the two strands forms an anti‐parallel *β*‐sheet, and of other regions (blue) of the Hsp110 NBD that are also involved in contacts with the HSp70 linker; the remaining parts of the two proteins are rendered in transparency for clarity; (E) Same as in (D), with highlight of the hydrophobic residues in space‐filing representation; (F) Segments of HspA8 and HspA8 involved in the contacts in panels (D) and (E). Structures have been obtained with AlphaFold 3 [18] and relaxed with Rosetta [93, 94, 95].

The Hsp70‐Hsp110 heterodimer is reminiscent of, but distinct from, the homodimeric Hsp70‐Hsp70 associations described previously [21, 88, 89, 90], whose functional relevance remains unresolved [91]. The crystallographic structure of the Hsp70‐Hsp110 complex [87] for bovine Hsp70 HspA8 with yeast Hsp110 Sse1, together with AlphaFold [18] predictions for the bovine Hsp70–Hsp110 heterodimer (Fig. 5A), shows Hsp70 (HspA8) in an ADP‐bound state, with a closed SBD undocked from the NBD (Fig. 5B), whereas Hsp110 (HspA4) adopts an ATP‐bound like conformation, with an open SBD docked onto its NBD (Fig. 5C). Remarkably, although the interdomain linker of Hsp70 is disordered and exposed in monomeric ADP‐bound Hsp70, in the Hsp70–Hsp110 heterodimer it adopts an antiparallel *β*‐strand conformation paired with a prelinker segment of the Hsp110 NBD (Fig. 5D). A closer inspection reveals that this hydrophobic Hsp70 linker is buried in a hydrophobic cradle (Fig. 5E) formed by the Hsp110 prelinker and sequence‐distal regions of its NBD (Fig. 5F), which include two arginine and one lysine residues contributing via the aliphatic portions of their side chains. Sequestration of the linker in this cradle could attenuate the allosteric signal associated with ADP release and ATP rebinding, which would otherwise promote linker docking onto the NBD, separation of the two SBD subdomains and their docking onto the ATP‐bound NBD [92]. Taken together, these observations place the reversible interaction between the NBD of Hsp110 and that of Hsp70 at the core of a plausible mechanism of Hsp110 action, directly controlling both nucleotide exchange and the subsequent opening of the SBD of Hsp70 and hence substrate release, which may then occur only after a delay during which the bound Hsp110 could amplify the unfolding effect of entropic pulling by the bound Hsp70.

The Nucleotide Binding Domain, by definition, is where ATP binds and can be hydrolyzed, and its functional centrality naturally raises the question of how the Hsp110 ATPase cycle contributes to Hsp110 mechanism. Yet, the role of ATP hydrolysis by Hsp110 remains contentious. Hsp110s do intrinsically hydrolyze ATP [77, 96, 97], and this activity can be stimulated by interactions with Hsp70 [96], but the physiological relevance of this ATPase is unclear. Indeed, ATP hydrolysis by Hsp110 does not appear necessary to stimulate nucleotide exchange [97, 98] in Hsp70, nor does it markedly enhance Hsp70‐dependent disaggregation at low Hsp110 concentrations [77, 86]. When at higher concentrations, however, Hsp110 inhibits Hsp70‐mediated disaggregation [77, 99], and this inhibitory effect is even stronger for ATPase‐deficient yeast Hsp110 (Sse1) [77]. Because higher concentrations of Hsp110 are expected, by mass action, to stabilize the Hsp70‐Hsp110 complex, it has been proposed that ATP hydrolysis by Hsp110 is needed to drive dissociation of the heterodimer, in line with previous proposals [100]. Resolving this issue will require further work, including the possibility that ATPase activity is mechanistically important for some Hsp110 paralogs but dispensable for others.

The functional divergence between Hsp70 and Hsp110 correlates with their structural and sequence diversification. The linker between the NBD and the SBD no longer resembles the highly conserved motif typical of Hsp70s (e.g., ^391^EFSITD^396^ for bovine HspA4, instead of ^390^DLLLLD^395^ for bovine HspA8), possibly contributing to the loss of ATPase stimulation by J‐domains [17, 74, 92]. Substrates have also lost the ability to stimulate ATP hydrolysis by Hsp110 [77], limiting the protein‐binding capability of Hsp110s. Furthermore, the SBD of Hsp110s acquired long insertions and extensions in the *β*‐basket and the *α*‐helical lid, whose function is still unclear, although deletion of some of these extensions affects the Hsp70 ATPase cycle [99, 101]. Overall, changing an original Hsp70 to turn it into a modern Hsp110 required tinkering with the four basic requirements for a functional Hsp70: The structures and sequences of the NBD, SBD, and linker have been altered; the NBD ATPase activity, if relevant, is likely triggered by signals completely different from the ones that activate Hsp70s; the interactions with J‐domains have been abolished, as well as the ones with substrates, at least in part. For this last point, indeed, it should be noted that there are some discrepancies regarding the interaction between Hsp110 and substrates depending on substrate type, and certain substrates or peptides can bind Hsp110 differently than Hsp70 [102, 103].

As a side note, we recall that Hsp110s are not the sole potentiators of Hsp70‐mediated disaggregation. Most organisms, with the exception of the Metazoa and the majority of Archaea, possess a dedicated chaperone of the AAA+ Hsp100 family, whose evolution can be traced back to the bacterial homolog (ClpB) that cooperates with Hsp70 to optimize disaggregation. Indeed, Hsp70s possess an intrinsic, stand‐alone ability to disassemble and refold small inactive protein aggregates [35] with almost no effect upon the addition of ClpB [24, 26]. Yet, multiple Hsp70s can remain ineffective on large compact aggregates unless they are supplemented with ClpB, which alone is ineffective, but together dramatically improves the disaggregation/refolding reaction [104, 105]. It is thus possible that there is no definitive answer to whether the contribution of ClpB is stringently needed or unnecessary, most likely depending on the nature of the aggregates. Like the other members of the AAA+ family, functional Hsp100s are rings composed of six ATPase protomers. Their action, driven by ATP hydrolysis, results in the unfolding and disaggregation of their substrates by a mechanism that involves their complete or partial threading of polypeptide loops through their central pore. Structurally and mechanistically, Hsp100s could thus not be more different from Hsp110s. Yet, it is noticeable that in the cytosol of plants and fungi (where they are known as Hsp101 and Hsp104, respectively), and most protozoans, Hsp100s coexist with Hsp110, suggesting that evolution developed Hsp110 on top of a pre‐existing Hsp100‐based (ClpBs) disaggregating cochaperone. Their ubiquitous co‐occurrence for almost two billion years (with the loss of ClpB in Metazoa possibly about one billion years ago) [106] suggests that their combined action might confer a selective advantage, possibly by facilitating the recruitment of the ClpB analogue, Hsp104, on aggregates [105] and/or by expanding the repertoire of aggregate typologies that the Hsp70 system can successfully solubilize.

While Hsp110s are among the best‐characterized Hsp70‐derived paralogs in eukaryotic cells, there are several others that are known but mechanistically partially or completely untested, such as HspA12A/B and HspA14, which lack the *α*‐helical lid, and HspA13, whose SBD is completely missing. Functionally, HspA14 corresponds to yeast Ssz1, a member of the Ribosome‐Associated Complex (RAC) [107], which, together with the dedicated JDP zuotin, recruits the cytosolic Hsp70 paralog Ssb to act on nascent proteins. The interaction of Ssz1 with Ssb is partially mediated by their NBDs [108]. The function of HspA12 is presently unknown although, curiously, in bivalves, the HspA12A/B genes have undergone a massive expansion being counted in the tens per genome [109, 110, 111]. It is unclear which diverse stresses in bivalves have favored such extensive paralog expansion, but the sessile, filter‐feeding lifestyle of many Bivalvia, which concentrates pathogens, metals, and other potent cellular stressors, suggests that HspA12 may help buffer these insults, possibly in partnership with canonical Hsp70s.

From a more general perspective, a simple exploration of the Uniprot [56] database reveals an extremely diverse repertoire of Hsp70 derivatives also in eukaryotes. Once again (and expectedly) the vast majority of them are easily recognizable by the presence of a well‐conserved NBD, while the SBD has undergone most changes.

## Evolutionary and functional perspectives on the emergence of DnaK derivates

**Fig. 6:**
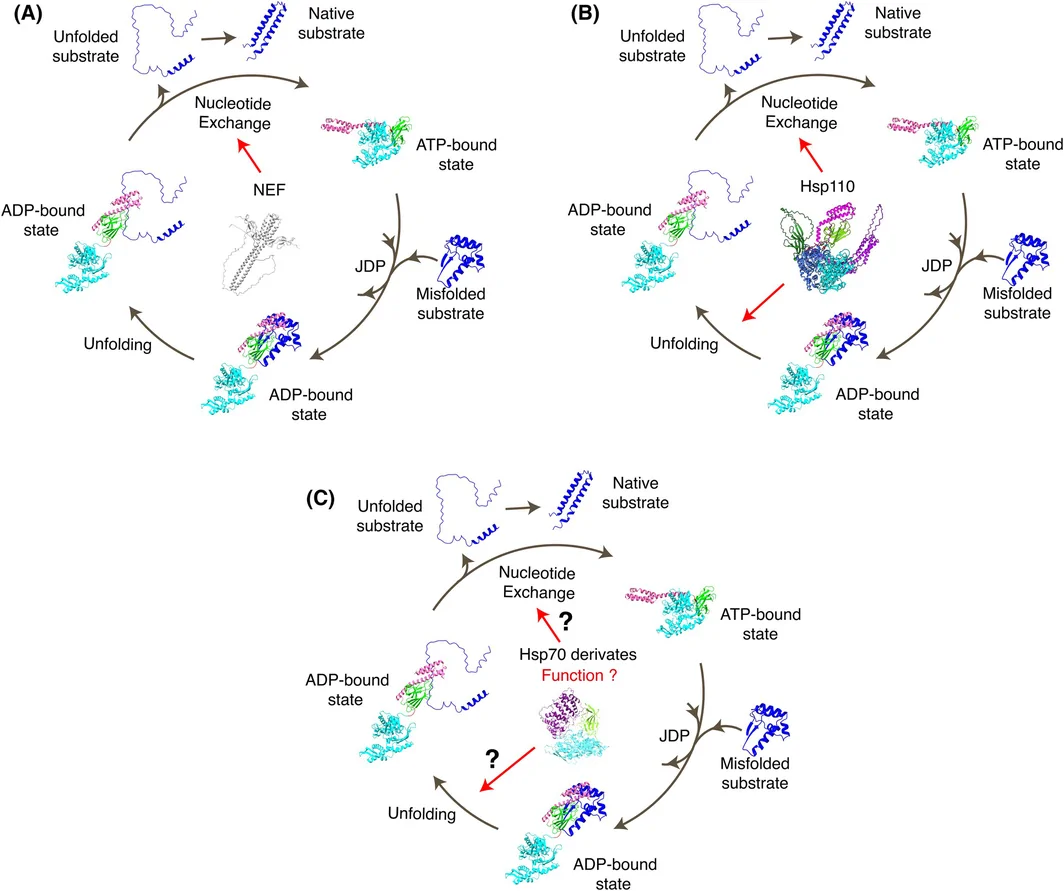
Evolutionary and mechanistic models of the Hsp70 chaperone cycles. (A) Role of NEFs in the Canonical Hsp70 system (DnaK‐type). The ATP‐dependent folding cycle of prokaryotic Hsp70s is regulated by J‐domain proteins (JDPs), which target misfolded substrates, stimulate ATP hydrolysis by Hsp70, and cause the locking of the Substrate Binding Domain (SBD) on the substrate. This is followed by substrate unfolding and then by nucleotide exchange, possibly stimulated by Nucleotide Exchange Factors (NEFs) such as GrpE, Fes1, or Bag‐domain proteins that promote ADP release and rebinding of ATP, causing the opening of the SBD and release of the unfolded substrate that can now fold to the native state. (B) Role of eukaryotic Hsp110‐like proteins. The binding of Hsp110 to Hsp70 involving extensive NBD‐NBD contacts, plays the double role of a NEF and of an enhancer of the disaggregating/unfolding activity of Hsp70; (C) Speculative roles of Hsp70 derivates in bacteria. Duplicated or specialized Hsp70s retain the conserved Nucleotide Binding Domain (NBD) but display additional variable domains often of unknown function. These variants may have diverged from canonical Hsp70s to act as co‐chaperones helping Hsp70 to perform specialized or regulatory roles within the bacterial proteostasis network. While the analogy of Hsp110 suggests that some of these functions could be to act as NEFs or as unfolding/disaggregation enhancer, their precise mechanistic contribution remains presently unknown.

Hsp70 cochaperones play a crucial role to regulate its functional cycle. Canonical Nucleotide Exchange Factors (NEFs), such as GrpE in prokaryotes, Fes1‐like, and/or Bag‐domain containing proteins in eukaryotes, control the rate of SBD opening with the consequent release of the unfolded substrate that can then refold to its native state (Fig. 6A). In eukaryotes, Hsp110 has emerged from the duplication and subsequent divergence of the first eukaryotic Hsp70s. While initially identified as a NEF for Hsp70 by means of interactions extensively involving NBD‐NBD contacts, Hsp110 has also been recognized as an enhancer of Hsp70‐mediated disaggregation (Fig. 6B). Based on comparative evidence from Hsp70 evolution, structure, sequence, and function, together with insights from Hsp110, we propose here that some bacterial DnaK derivates that are recognizable only through their canonical NBD may interact transiently with DnaK via NBD–NBD contacts. By coupling their ATPase cycles to DnaK's hydrolysis‐ or exchange‐driven conformational changes, some, but not necessarily all, of these derivatives could act as co‐chaperones, in some cases functioning as NEFs or enhancing disaggregation (Fig. 6C). Future work is needed to test this idea.

The widespread presence and the extreme diversity of these proteins raise intriguing questions about what ecological pressures and cellular lifestyles promoted their emergence, conservation, and, in some cases, horizontal transfer to other prokaryotes. With the sole exception of HscA, which assists iron–sulfur cluster assembly [52], and to a much lesser degree HscC [53], bacterial DnaK derivates remain a largely unexplored ‘dark matter’ of the chaperone network.

The expansion of DnaK‐derived proteins, especially in prokaryotes with more complex proteomes, may have been favored in mesophilic lineages and in environments that fluctuate over time [50, 112]. In extremophiles, proteins face strong selection for stability under extreme but relatively constant conditions [113, 114, 115, 116]. By contrast, mesophiles live in milder yet continually changing conditions, which may limit how tightly sequences can be optimized [117, 118]. This can leave proteins more vulnerable to misfolding stress [1], creating selective pressure for additional Hsp70‐based functions. In this view, canonical DnaK is the ancestral, broadly acting unfoldase/refoldase, whereas derived systems, such as the eukaryotic Hsp110s, evolved in specific contexts to add regulation and specialization to the Hsp70 network, supporting proteostasis across diverse and increasingly challenging lifestyles.

## Author contributions

MER, PG, and PDLR were involved in conceptualization, writing original draft, and review and editing.

## Conflict of interest

The author declares that he has no known competing financial interests or personal relationships that could have appeared to influence the work reported in this paper.

## Supporting information


**Data S1.** JDPs, GrpE, ClpB and Hsp70‐like genes in 130 phylogenetically distant prokaryotic genomes and AlphaFold pLDDT, ipTM and pTM values.

## Data Availability

All data are available in the main text or in the supporting information.
